# Diaphragmatic stripping versus full-thickness diaphragmatic resection in cytoreductive surgery: a meta-analysis of the current evidence

**DOI:** 10.1007/s00423-025-03611-0

**Published:** 2025-01-25

**Authors:** Maria Chara Stylianidi, Sascha Vaghiri, Alireza Pandkhahi, Sultan Kazziha, Ward Al Akeel, Wolfram Trudo Knoefel, Dimitrios Prassas

**Affiliations:** 1 Department of Surgery (A), Medical Faculty and University Hospital Duesseldorf, Heinrich-Heine-University, Moorenstr. 5, 40225 Duesseldorf, Germany; 2 Department of Surgery, Katholisches Klinikum Essen, Philippusstift, Teaching Hospital of Duisburg-Essen University, Huelsmannstr. 17, 45355 Essen, Germany; 3 Medical Research School Duesseldorf, Heinrich-Heine-University Duesseldorf, Moorenstr. 5, 40225 Duesseldorf, Germany; 4 Department of Surgery (A), Medical Faculty and University Hospital Duesseldorf, Heinrich-Heine-University, Moorenstr. 5, Bldg. 12.46, 40225 Duesseldorf, Germany

**Keywords:** Peritoneal carcinomatosis, Cytoreductive surgery, Diaphragmatic surgery, Diaphragmatic stripping, Full thickness diaphragmatic resection, Pulmonary complications

## Abstract

**Purpose:**

The primary objective was to compare the intra- and postoperative outcomes of diaphragmatic stripping versus full-thickness diaphragmatic resection in patients with peritoneal carcinomatosis who underwent cytoreductive surgery.

**Methods:**

According to the PRSIMA guidelines, a comprehensive literature search was conducted for studies comparing postoperative pulmonary complications as well as intra- and postoperative outcomes of diaphragmatic stripping versus full-thickness diaphragmatic resection in patients with peritoneal carcinomatosis necessitating cytoreductive surgery. Data from eligible studies were extracted, qualitatively assessed, and included in a meta-analysis. Odds ratios (ORs) and standardized mean differences (SMDs) with 95 per cent confidence intervals were calculated.

**Results:**

Ten studies with 1325 patients were included in this meta-analysis. Diaphragmatic stripping was associated with lower incidence of pleural effusion (OR 0.47, 95% CI 0.35–0.63, *p* < 0.00001) and pneumothorax (OR 0.52, 95% CI 0.35–0.78, *p* = 0.002), less severe postoperative complications (Clavien-Dindo Grade ≥ 3) (OR 0.43, 95% CI 0.30–0.63, *p* < 0.0001), and shorter duration of surgery (SMD -0.31, 95% CI -0.54 – -0.08, *p* = 0.007). No significant differences were observed in postoperative subdiaphragmatic abscess occurrence, intraoperative blood loss, hospital- and ICU-stay, and 90-day mortality.

**Conclusions:**

Diaphragmatic stripping leads to a significantly lower rate of postoperative pulmonary and severe complications compared to diaphragmatic full-thickness resection, while oncological outcomes do not appear to be worse. Larger trials with standardized study protocols and long-term survival data are needed to validate the results presented here.

**Supplementary Information:**

The online version contains supplementary material available at 10.1007/s00423-025-03611-0.

## Introduction

Peritoneal carcinomatosis is the term used to describe malignancies of the peritoneal surface and includes primary peritoneal cancers such as mesothelioma, peritoneal manifestations of other gastrointestinal and genital tumours, and sarcomas [1]. A novel approach to the treatment of peritoneal carcinomatosis, consisting of parietal and visceral peritonectomy followed by hyperthermic perioperative chemotherapy (HIPEC) with the aim of complete cytoreduction, was introduced by Sugarbaker in the 1980s [2]. Sugarbaker`s cytoreduction approach is associated with improved survival despite its high 30-day mortality and morbidity rates of 0.8–4% and 22–55%, respectively [3]. Therefore, multi-organ debulking surgery, frequently including diaphragmatic surgery due to direct diaphragmatic invasion, is often performed to achieve potential complete cytoreduction in patients with peritoneal carcinomatosis [4, 5]. Depending on the volume, distribution and depth of infiltration of metastatic lesions, several techniques have been proposed for resection of diaphragmatic disease involvement. These procedures range from electrocoagulation and vaporisation to more extensive and significant procedures such as diaphragmatic stripping, which is performed when superficial disease extension is found, and full-thickness diaphragmatic resection (DFTR), which is performed when all or part of the muscle thickness is involved [6].

Although several studies have been published in recent years, mostly from European and US centres, focusing mainly on ovarian cancer patients, there are few studies comparing diaphragmatic stripping and total diaphragmatic resection for pulmonary complications including pleural effusion and pneumothorax and outcomes such as operation time, hospital-and ICU stay [7–11]. Therefore, the aim of this study was to perform a meta-analysis of studies comparing the intraoperative features, the postoperative complications, and recovery of diaphragmatic stripping and diaphragmatic full-thickness resections in patients who underwent cytoreductive surgery with or without HIPEC for peritoneal carcinomatosis.

## Materials and methods

This meta-analysis was carried out using the current PRISMA (Preferred Reporting Items for Systematic Reviews and Meta-Analyses) checklist and the Cochrane Handbook for Systematic Reviews of Interventions [12, 13].

### Eligibility criteria and group definition

All studies that compared the postoperative clinical outcomes of patients who underwent diaphragmatic stripping versus full-thickness diaphragmatic resection for peritoneal carcinomatosis were considered eligible. To avoid heterogeneity, studies were selected for final analysis if they included patients with peritoneal carcinomatosis and diaphragmatic involvement in gastrointestinal (GI) and gynecological malignancies.

Outcomes of particular interest were postoperative complications such as pleural effusion, pneumothorax subdiaphragmal abscess, and Clavien-Dindo Grade ≥ 3 complications. Other analyzed parameters were surgery duration, intraoperative bleeding, ICU, hospital stay, and 90-day mortality. Studies had to report at least one of the outcomes listed above to be included in the analysis. All types of published studies involving human participants within the defined inclusion criteria were considered for further selection and analysis (e.g. randomized controlled trials (RCTs), and prospective or retrospective comparative cohort studies). Disagreements or differing conclusions in the selection of studies were resolved either by consensus or by consultation with an independent third author (D.P.).

### Literature search

A literature search was conducted independently by two authors (S.V. and D.P.), who systematically identified all relevant studies up to August 2024 in Pubmed (Medline), the Cochrane Central trials register and the google scholar databases. There were no language or time restrictions. The following search terms were used in combination with the Boolean operators AND or OR: "Diaphragmatic resection", "stripping “, „full thickness resection", "CRS" "cytoreductive surgery “,"HIPEC". Furthermore, the reference list of the retrieved articles (including systematic reviews, case reports, editorials or experimental studies that were initially excluded) was manually reviewed to identify potential citations for analysis. If there were duplicates or overlaps between articles published by the same institution and by the same author, the most recent study was included.

### Data extraction and outcome measures

A self-developed electronic data extraction sheet was used independently by four authors (A.P., S.K., W.A., and M.C. S.) to enter all relevant data from studies within the eligibility criteria. Study, patient, and operative-specific information included country of origin, year of publication, study design, enrolment period, number of patients enrolled per group and their demographics (age, sex, body mass index (BMI), (American society of anesthesiologist) ASA class), indication for surgery, type of procedure, duration of surgery, and intraoperative blood loss. The primary endpoints were major postoperative morbidity defined by Dindo-Clavien Grade ≥ 3, postoperative complications and specifically the rate of pleural effusion, pneumothorax and subdiaphragmatic abscess formation. The secondary outcome analysis included the following objectives: duration of surgery, hospital stay, ICU stay and 90-day-mortality. Discrepancies in data extraction were resolved by consensus or reassessment by an independent third author (S.V.) to ensure consistency and accuracy.

### Quality and certainty assessment

The risk of bias in the included non-randomized studies was assessed independently by three authors (A.P., S.K., and W.A.) using the ROBINS I criteria [14]. In short, this recommended tool classifies non-randomized trials as low to high risk for bias using signal questions derived from seven potential different domains of bias at three time points in each study: pre-intervention (confounding and selection of participants), at intervention (classification of interventions), and post-intervention (bias due to deviations from the intended interventions, missing data, measurement of outcomes, and selection of the reported outcome). The reviewers were not blinded to the study authors. Disagreements in the study bias assessment were discussed and resolved by consensus or consultation of an independent co-author (S.V.). The Grading of Recommendations, Assessment, Development, and Evaluation (GRADE) methodology was applied to adequately document the strength and certainty of evidence using four levels for significant outcome parameters (high, moderate, low, and very low) [15, 16].

### Statistical analysis

Statistical analysis was performed using RevMan software (version 5.3; Copenhagen: The Nordic Cochrane Centre, The Cochrane Collaboration, 2014). Paired meta-analyses were performed. Summary treatment effect estimates with 95% confidence intervals (CIs) were calculated for each outcome of interest. In the case of dichotomous outcomes, the odds ratio (OR) was used as the effect measure, while standardized mean differences (SMDs) were calculated for continuous parameters. For continuous variables, the method proposed by Luo et al. was used to convert the available data from medians and interquartile range (IQRs) into means and standard deviations [17]. Of note, continuous outcomes were expressed in minutes (duration of surgery) or days (duration of hospital stay, intensive care unit (ICU) stay). The degree of heterogeneity among the included studies was interpreted using the Cochrane Q test (chi-squared test; chi2) and the inconsistency measure (I^2^) as follows: 0%−40% low heterogeneity and may not be important, 30%−60% moderate heterogeneity, 50%−90% substantial heterogeneity, > 75% high heterogeneity [8]. Summary estimates were calculated using a fixed-effects method if heterogeneity was low or moderate (I^2^ < 50%). If the I^2^ was > 50%, the randomized model was used. Subgroup analyses to examine heterogeneity of results were performed when appropriate. Due to the small number of studies included in the meta-analysis, publication bias tests and funnel plots were not performed. P-values < 0.05 of pooled data were considered significant.

## Results

### Study and patient characteristics

**Fig. 1:**
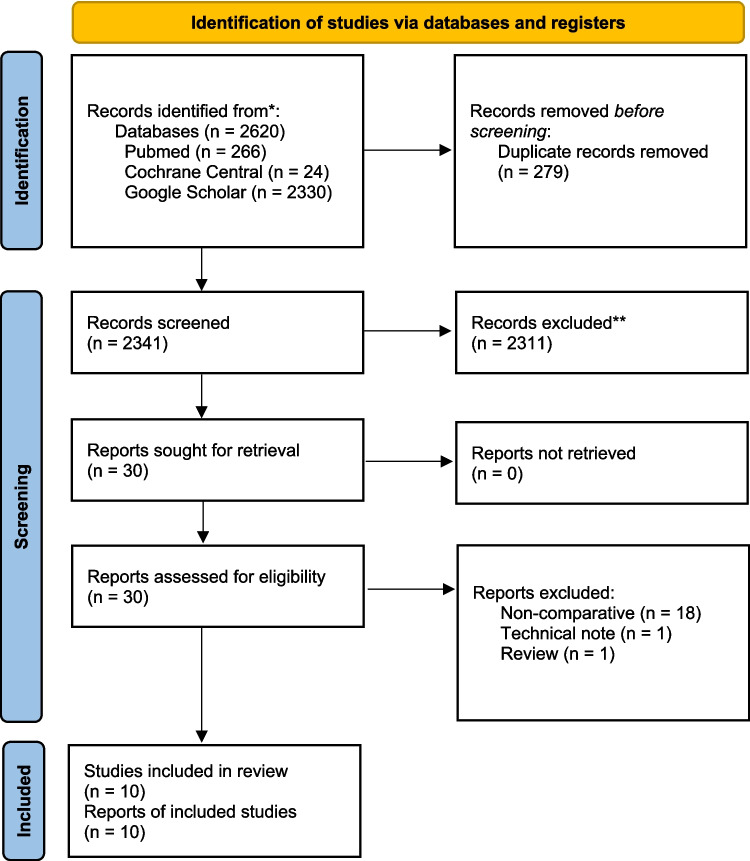
PRISMA diagram of study identification and selection for review analysis

The initial database query yielded in 2620 results. After critical review and selection of the included reports, 30 full-text articles were screened for eligibility and 10 non-randomized monocentric-studies were included in the final qualitative and quantitative data analysis. The detailed selection process is depicted in the PRISMA Flowchart (Fig. 1).

A total of 1325 patients (Stripping: n = 946, Full-Thickness Diaphragmatic Resection: n = 379) form the final study cohort [18–27]. Six studies originated from Europe [18–20, 23, 25, 27], one from USA [24], one from Australia [22], one from China [21] and one from Japan [26]. The study enrolment period was from September 1993 to October 2019. All 10 studies were single-centre studies [18–27]. Nine studies were retrospective studies [18–26] while the study by Pathiraja et al. was conducted prospectively [27]. The reported male to female ratio in nine studies was 74:747 [18–21, 23–27], while Singh et al. provided no data regarding the numbers of male and females patients [22]. This male to female ratio reflects the fact that most of the trials in this meta-analysis included patients with ovarian cancer at FIGO III/IV stage [18, 20, 21, 23, 25–27]. All studies included patients who underwent multi-visceral surgery, but only five included detailed information about the surgical procedures performed [18, 20, 21, 23, 27]. The study, patient-and operative characteristics are summarized in detail in Tables 1 and 2.

**Table 1:** Study characteristics and protocols

Author	Year	Origin	Study period	Study design	Exclusion Criteria	Total sample size	Tumour entity (%)	Surgical Technique for Defect Closure in full-thickness resection
Zapardiel et al. [18]	2011	Italy	January 2001-December 2008	Retrospective	Prior attempt of surgical cytoreduction at another institution, stage IIIC disease based on nodal metastases alone, histology consistent with non-epithelial ovarian malignancies or borderline tumors, neo-adjuvant CTX	112	Ovarian Cancer (100)	Running no. 1-Prolene suture on CT-1 needles
Craus-Miguel et al. [19]	2021	Spain	January 2011-October 2019	Retrospective	Non-resectable disease, thoracic surgery in the same procedure, ECOG score > 1, lacking follow-up data	88	Gynecological:51 (58) Colon:20 (23) Appendix:12 (14) Mesothelioma:2 (2) Gastric:2 (2) Carcinosarcoma:1 (1)	Running polypropylene 0 suture
Pounds et al. [20]	2018	United Kingdom	August 2007—February 2016	Retrospective	NA	69	Ovarian Cancer (100)	Running polypropylene 0 suture
Ye et al. [21]	2017	China	January 2009- August 2015	Retrospective	NA	150	Ovarian Cancer (100)	NA
Singh et al. [22]	2019	Australia	1996–2018	Retrospective	Patients undergoing redo surgery, incomplete cytoreduction, and other than adenocarcinoma, HAMNs, LAMNs and mesothelioma	1230	Colorectal:114 (21.6) HAMNs:181 (34.3) LAMNs: 178 (33.7) Mesothelioma:55 (10.4)	NA
Soleymani et al. [23]	2016	United Kingdom	April 2009—November 2013	Retrospective	Pre-operative: lung metastases, 3 or more liver segments involvement, disease progression following chemotherapy Intra-operative: diffuse small bowel serosal deposits, porta hepatis encasement	100	Ovarian Cancer (100)	Running 0 PDS suture
Sullivan et al. [24]	2020	United States	March 2007—June 2018	Retrospective	NA	171	LAMNs:88 (21) Mesothelioma:22 (5) Colorectal:119 (29) Ovarian: 17(4) Appendiceal cancer:86 (21) Upper GI:35 (9) Hepatobiliary:20 (5) Other:22 (6)	Running or interrupted prolene sutures
Tsolakidis et al. [25]	2010	Belgium	April 2009—March 2012	Retrospective	Poor general condition (e.g. > 80 years old) making a “maximal surgical effort” to no residual tumor impossible. Intrahepatic multiple metastases larger than 2 cm. Extra-abdominal metastatic disease (> 2 cm diameter), excluding supraclavicular and inguinal node metastases. Metastatic disease, > 2 cm diameter at the level of the porta hepatis. Metastatic disease, > 2 cm diameter at the level of the superior mesenteric artery. Extensive serosal invasion (plaques) of the intestines necessitating multiple bowel resections totalling > 1.5 m of bowel resection	38	Ovarian cancer (100)	Full-thickness sutures of polyglactin
Terauchi et al. [26]	2010	Japan	September 1993—December 2007	Retrospective	NA	68	Ovarian Cancer (100)	Absorbable sutures (PDS)
Pathiraja et al. [27]	2013	United Kingdom	November 2006—March 2009	Prospective	ASA > 3, performance status > 2 and metastases in the lungs and/or in 3 or more liver	42	Ovarian Cancer (100)	Continuous 0 PDS full thickness suture

*CTX* Chemotherapy; *NA* Not applicable; *HAMNs* High-grade appendiceal mucinous neoplasms; *LAMNs* Low-grade appendiceal mucinous neoplasms; *PDS* Polydioxanone; *ASA* American Society of Anesthesiology

**Table 2:** Demographic data and characteristics of the included patients

Author	Groups	No. of patients	Age (years) mean/SD	Gender (M/F)	BMI (kg/m^2^) mean/SD	ASA score (%)	FIGO Stage*(%)
Zapardiel et al. [18]	DS	79	56.6 ± 10.7	0/79	NA	I:10 (12.6) II:21 (26.6) III:47(59.5) IV:1(1.3)	IIIC:57 (72.2) IV:22 (27.8)
	DFTR	33	51.2 ± 11.8	0/33	NA	I:5 (15.1) II:16 (48.5) III:12 (13.4) IV:0 (0)	IIIC: 21 (63.6) IV:12 (36.4)
Craus-Miguel et al. [19]	DS	56	59.16 ± 15.22	10/46	NA	I:7 (12.5) II:41 (73.2) III:8 (14.3)	NA
	DFTR	32	60.63 ± 12.03	5/27	NA	I:7 (12.5) II:41 (73.2) III:8 (14.3)	NA
Pounds et al. [20]	DS	38	60.3 ± 9.5	0/38	NA	NA	IIIB:2 (5.3) IIIC:29 (76.3) IV:7 (18.4)
	DFTR	31	58.6 ± 10.1	0/31	NA	NA	IIIB:1 (3.2) IIIC:15 (48.4) IV:15 (48.4)
Ye et al. [21]	DS	124	NA	0/124	NA	NA	NA
	DFTR	26	NA	0/26	NA	NA	NA
Singh et al. [22]	DS	428	Colorectal:49 Mesothelioma:56 HAMNs:53 LAMNs:55	NA	NA	ASA II: Colorectal:2 (6) Mesothelioma:10 (26) HAMNs: 68 (40.2) LAMNs:44 (31)	NA
	DFTR	76	Colorectal:61 Mesothelioma:50 HAMNs:59 LAMNs:55	NA	NA	ASA II: Colorectal:21 (27) Mesothelioma:7 (44) HAMNs:2 (16.7) LAMNs:5 (14)	NA
Soleymani et al. [23]	DS	64	63 ± 13.08	0/64	NA	NA	IIIC:54 (84) IV:10 (16)
	DFTR	36	64 ± 11.76	0/36	NA	NA	IIIC:23 (64) IV:13 (36)
Sullivan et al. [24]	DS	49	55 ± 3.50	15/34	25 ± 1.34	II:13 (27) III:35 (71) IV:1 (2)	NA
	DFTR	105	56 ± 3.93	44/61	27 ± 1.50	II:13 (12) III:81 (77) IV:11 (11)	NA
Tsolakidis et al. [25]	DS	31	56 ± 11.58	0/31	NA	NA	IIIB:4 (13) IIIC:23 (74) IV:4 (13)
	DFTR	7	53 ± 6.84	0/7	NA	NA	IIIB:0 (0) IIIC:4 (57) IV:3 (43)
Terauchi et al. [26]	DS	56	NA	0/56	NA	NA	NA
	DFTR	12	NA	0/12	NA	NA	NA
Pathiraja et al. [27]	DS	21	64	0/21	NA	I-II:21 (100)	IIIc:20 (95) IV:1 (5)
	DFTR	21	63.5	0/21	NA	I-II:21 (100)	IIIc:14 (66) IV:7 (34)

^*^only studies with ovarian cancer

*DS* Diaphragmatic Stripping; *DFTR* Diaphragmatic full-thickness resection; *BMI* Body Mass Index; *ASA* American Society of Anesthesiology; *NA* Not applicable; *HAMNs* High-grade appendiceal mucinous neoplasms; *LAMNs* Low-grade appendiceal mucinous neoplasms; *FIGO* Federation of Gynecology and Obstetrics

### Study quality and risk of bias

**Fig. 2:**
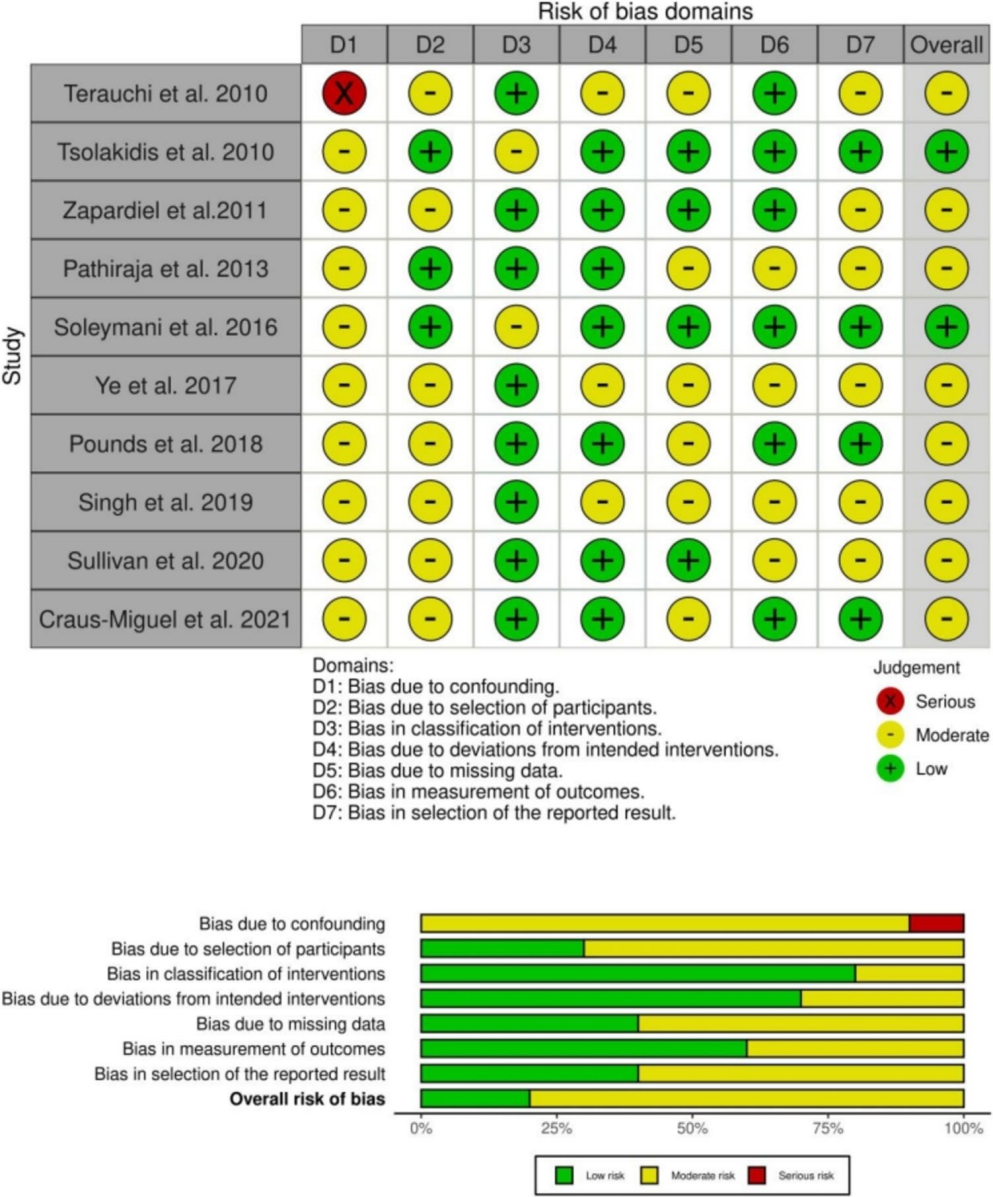
Risk of bias summary and graphical visualization of the included studies based on ROBINS-I-tool

With one exception [27], all included studies were retrospective studies [18–26]. The overall risk of bias according to the ROBINS-I tool was moderate (Fig. 2). The most limiting factors were beside lack of randomization, bias due to missing data and selection of the reported results. The quality of evidence for the significant primary and secondary outcomes ranged between very low and moderate with respect to the GRADE criteria.

### Outcome analysis

#### Primary endpoints

##### Pleural effusion

**Fig. 3:**
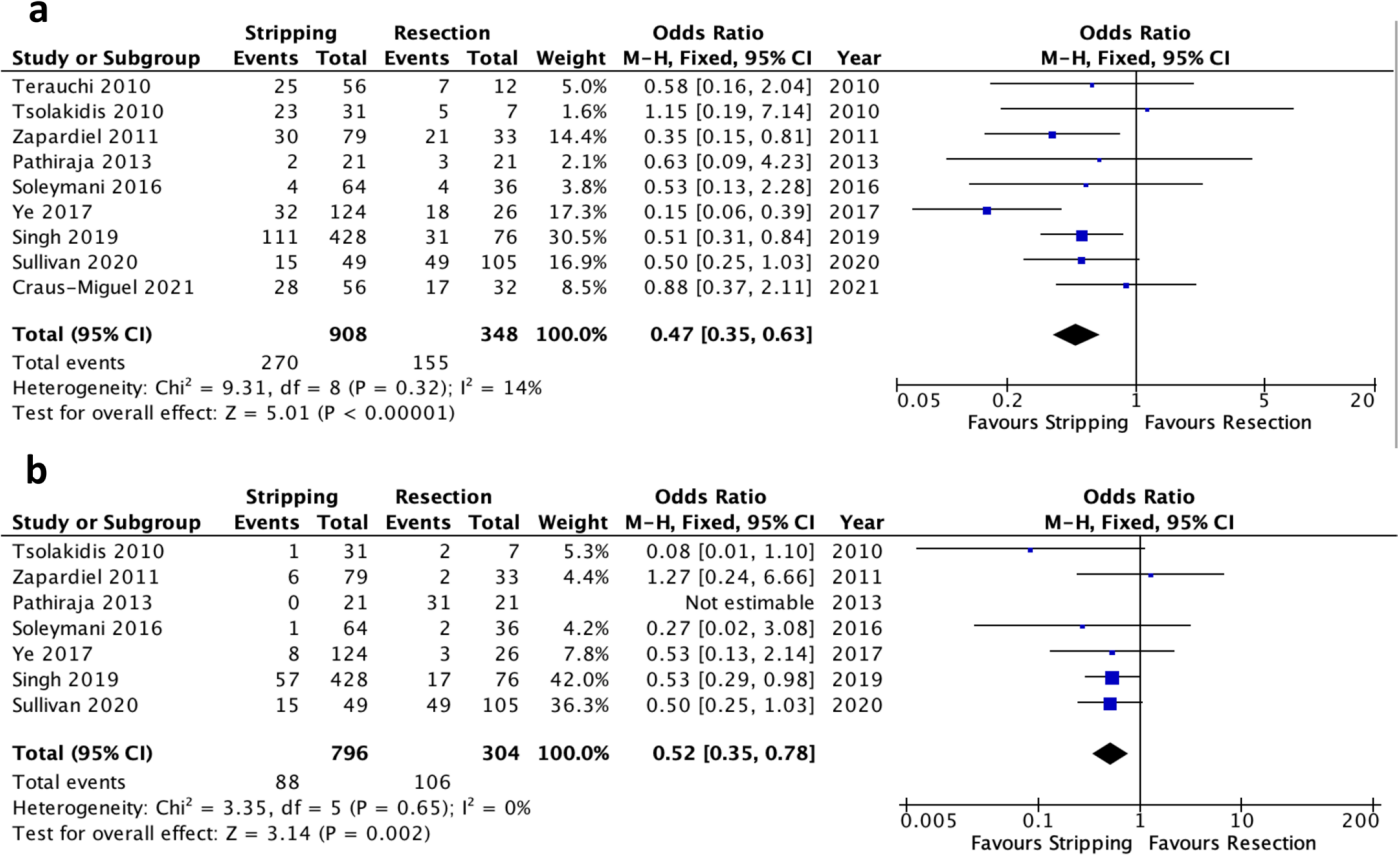
Forest plots of significant pulmonary outcomes: (**a**) pleural effusion, (**b**) pneumothorax

Pleural Effusion was reported in nine of the 10 included studies [18, 19, 21–27]. Meta-analysis of the pooled data revealed a significantly lower incidence of pleural effusion in the diaphragmatic stripping group compared to the full-thickness diaphragmatic resection cohort (OR 0.47, 95% CI 0.35–0.63, *p* < 0.00001). Notably, the level of heterogeneity was low (I^2^ = 14%, Chi^2^ test: *p* = 0.32) (Fig. 3a). The certainty of evidence was moderate (Table suppl. 1).

##### Pneumothorax

Pneumothorax was reported in seven of the 10 included studies [18, 21–25, 27]. Patients who underwent diaphragmatic stripping had a significantly lower incidence of pneumothorax than patients who underwent full-thickness diaphragmatic resection (OR 0.52, 95% CI 0.35–0.78, *p* = 0.002). The degree of heterogeneity was low (I^2^ = 0%, Chi^2^ test: *p* = 0.65) (Fig. 3b) with a moderate evidence level according to GRADE (Table suppl. 1).

##### Severe postoperative morbidity (Clavien-Dindo Grade ≥ 3)

**Fig. 4:**
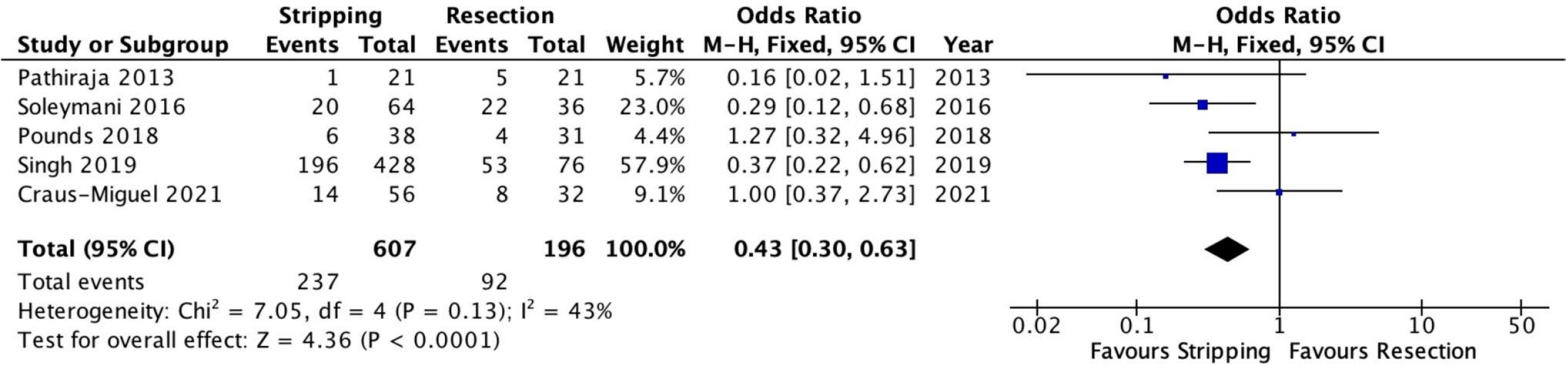
Forest plot of severe postoperative morbidity (Clavien-Dindo Grade ≥ 3)

Postoperative complications defined as Clavien-Dindo Grade ≥ 3 were reported in five studies [19, 20, 22, 23, 27]. There were significantly fewer reported Clavien-Dindo Grade ≥ 3 complications in patients who underwent diaphragmatic stripping compared to the full-thickness diaphragmatic resection group (OR 0.43, 95% CI 0.30–0.63, *p* < 0.0001). However, a moderate heterogeneity level was observed (I^2^ = 43%, Chi^2^ test: *p* = 0.13) (Fig. 4) with a moderate grade of evidence (Table suppl. 1).

##### Subdiaphragmatic abscess

**Fig. 5:**
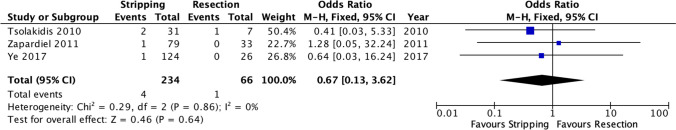
Forest plot of non-significant primary endpoints: subdiaphragmatic abscess

Subdiaphragmatic abscess was reported in three of the 10 included studies [18, 21, 25]. In these three studies there was no significant difference between the two groups (OR 0.67, 95% CI 0.13–3.62, *p* = 0.64). Moreover, a low heterogeneity degree was observed (I^2^ = 0%, Chi^2^ test: *p* = 0.86) (Fig. 5).

#### Secondary endpoints

##### Significant secondary endpoints: Surgery duration

**Fig. 6:**
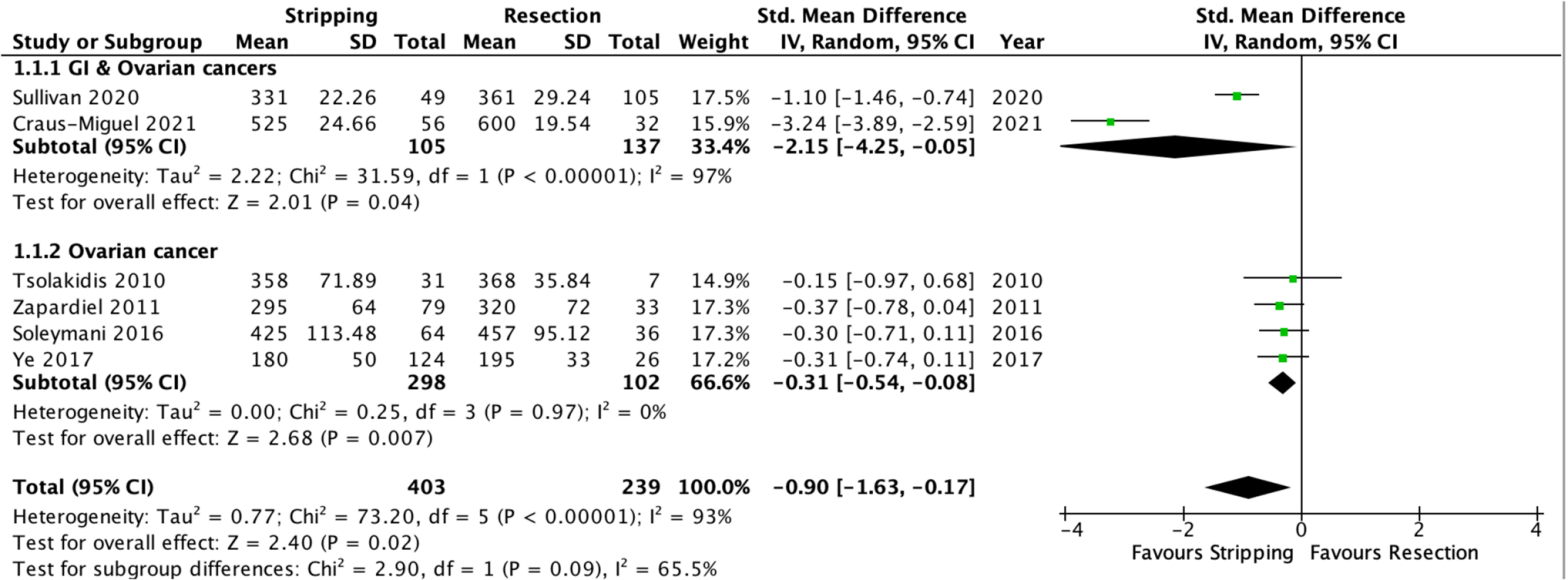
Forest plot of significant secondary endpoints: surgery duration

Surgery duration was reported in six studies [18, 19, 21, 23–25] The results of the secondary outcomes analysis revealed a significantly shorter surgery duration (SMD −0.90, 95% CI −1.63 – −0.17, *p* = 0.02) in the diaphragmatic stripping group. The same pattern was observed in the subgroup analysis of ovarian cancer studies [18, 21, 23, 25]. However, heterogeneity was substantially high (I^2^ = 93%, Chi2 test: p = 0.00001) (Fig. 6).

The source of heterogeneity was identified in studies including both GI and ovarian cancer cases [19, 24]. Four studies [18, 21, 23, 25] exclusively analyzed patients with ovarian malignancy. In this group diaphragmatic stripping still resulted in a significantly shorter duration of the procedure compared to the full-thickness resection technique (SMD −0.31, 95% CI −0.54 – −0.08, *p* = 0.007) with a low level of heterogeneity (I^2^ = 0%, Chi2 test: p = 0.97). Of note, the GRADE of evidence was very low for this outcome (Table suppl. 1).

##### Non-significant secondary endpoints

The 90-day mortality was reported in five studies [19, 20, 23, 24, 27], ICU-stay in three studies [18, 19, 25], intraoperative blood loss in five studies [18, 21, 23, 24, 27] and hospital stay also in six studies [18, 19, 23–25, 27]. Meta-analysis of the pooled data revealed no significant difference between diaphragmatic stripping and full-thickness diaphragmatic resection for the above-mentioned secondary endpoints (Table 3).

**Table 3:** Non-significant secondary outcomes

Outcomes	No. of included studies	No. of included patients		SMD/OR [95% CI]	P-value	Heterogeneity Level	
		Diaphragmatic Stripping	Full-Thickness Diaphragmatic Resection			I^2^ (%)	*P*-value
90-day mortality (days)	5 [19, 20, 23, 24, 27]	228	225	1.03 (0.30—3.50)	0.96	0	0.90
ICU-Stay (days)	3 [18, 19, 25]	166	72	−0.04 (−0.32 – 0.24)	0.77	0	0.94
Intraoperative Blood Loss (ml)	6 [18, 21, 23, 24, 27]	337	221	−0.42 (−1.05 – 0.21)	0.19	90	< 0.00001
Hospital Stay (days)	6 [18, 19, 23–25, 27]	300	234	−0.19 (−0.65 – 0.27)	0.41	83	< 0.00001

*ICU* Intensive Care Unit; *OR* Odds ratio; *SMD* Standardized mean difference

## Discussion

The present study is, to our knowledge, the first meta-analysis of pooled postoperative and surgical outcomes of diaphragmatic stripping versus full-thickness diaphragm resection in patients with peritoneal carcinomatosis. The cumulative results of 10 included studies with 1325 patients showed a significant benefit of diaphragmatic stripping over full-thickness diaphragm resection in terms of postoperative pleural effusion, pneumothorax, postoperative complications Dindo-Clavien grade ≥ 3, operative time and intraoperative blood loss. Interestingly, hospital stay was reported to be longer in the diaphragmatic stripping group. However, no difference was observed in ICU stay, 90-day mortality and postoperative incidence of subdiaphragmatic abscess. All the.

study participants including the medical stuff and study assessors were blinded.

The present study, with 10 studies included [18–27], aims to compare the intra- and postoperative outcomes of diaphragmatic stripping versus full-thickness diaphragmatic resection in patients with peritoneal carcinomatosis during cytoreductive surgery. Of the 10 selected studies, seven included patients with advanced ovarian cancer [18, 20, 21, 23, 25–27] and three [19, 22, 24] included other tumour entities such as mesothelioma and gastrointestinal tumours. The cumulative results of 10 included studies [18–27] with 1325 patients showed a significant benefit of diaphragmatic stripping over full-thickness diaphragmatic resection in terms of postoperative pleural effusion, pneumothorax, postoperative complications Clavien-Dindo Grade ≥ 3, and operative time duration. However, no difference was observed in intraoperative blood loss, ICU-and hospital stay, 90-day mortality, and postoperative incidence of subdiaphragmatic abscess.

Cytoreductive surgery is a currently accepted treatment option for several tumour entities that can lead to peritoneal carcinomatosis, such as colorectal cancer, mesothelioma and ovarian cancer [28–30]. Up to 50% of patients with peritoneal carcinomatosis have diaphragmatic involvement requiring stripping or resection to achieve microscopic tumor clearance and cytoreduction [22, 31]. The fact that seven [18, 20, 21, 23, 25–27] of the 10 analysed studies [18–27] in our meta-analysis included only patients with advanced ovarian cancer reflects the tendency in the literature that diaphragmatic resection procedures are mostly studied in patients with gynecological malignancies. The reason for this could be that in the vast majority of patients with advanced ovarian cancer the diaphragm is affected on at least one side, with an incidence of 40–75% reported in the literature, so that diaphragmatic surgery had to be performed to achieve potential complete tumor removal [9, 10].

Postoperative pulmonary complications such as pleural effusion and pneumothorax are common after cytoreductive surgery when the diaphragm is involved [32–34]. Possible risk factors include diaphragmatic surgery itself regardless of the technique, liver mobilisation, pleural incision and release of VEGF or inflammatory mediators [8, 27, 32]. In our meta-analysis, postoperative pleural effusion was reported in nine of the 10 included studies [18, 19, 21–27]. Our results showed a lower incidence of pleural effusions in the diaphragmatic stripping group in comparison to the full-thickness diaphragmatic resection group. This finding is in line with a previously published meta-analysis in the same topic from Bogani et al. [35] and a review from Giannini et al. [36]. One reason for the higher incidence of pleural effusion in the full-thickness resection group could be the need to open the pleural cavity during this procedure [6, 37].

Regarding the incidence of pneumothorax, our meta-analysis showed a lower incidence in the diaphragmatic stripping group. This finding contradicts the meta-analysis by Bogani et al., which documented a similar rate of pneumothorax between the two groups. This discrepancy may be due to newly published studies that were included in our meta-analysis but not in the meta-analysis of Bogani et al. [35]. Bashir et al. state in their study that the incidence of postoperative pneumothorax is similar for the different diaphragmatic resection techniques and could be minimized by using the appropriate surgical method [9]. Based on Bashir's statement, we could explain this controversy by the different surgical approach and suture material used to close the diaphragmatic defect in the included studies (Table 2).

There is a debate in the published literature as to whether a chest tube should be placed during primary surgery and diaphragmatic resection in order to reduce the occurrence of pulmonary complications. In none of the studies included in our meta-analysis that reported pulmonary complications chest tubes were inserted electively [18, 19, 21–27]. No intraoperative chest tube placement was the practice in a study by Eisenhauer et al. Specifically, based on their results, this institute stated that the incidence of postoperative pleural effusion was too low to justify intraoperative chest tube placement [8]. The same practice of non-elective chest tube placement was carried out by Bashir et al. and Panici et al. [9, 38].

The development of subdiaphragmatic abscess as a postoperative complication was documented in three of the included studies [19, 22, 26]. The reported cumulative results suggest no significant difference between the two groups. This finding is consistent with the results of the meta-analysis of Bogani et al. [35]. However, this observation should be viewed critically as only three of the studies reported the rate of postoperative subdiaphragmatic abscess formation, and the small number of studies may be responsible for the non-significant result.

Six studies [18, 19, 21, 23–25] included operating time and five studies included intraoperative blood loss [18, 21, 23, 24, 27]. Diaphragmatic stripping was associated with shorter operation time, but no significant difference in intraoperative blood loss was found. These findings were confirmed in the subgroup-analyses of the ovarian cancer cohort with a notably low heterogeneity level. Moreover, the full-thickness diaphragmatic resection group showed a significant higher rate of severe complications in comparison to the diaphragmatic stripping group [19, 20, 22, 23, 27]. A possible explanation could be that full-thickness resection is a more complicated surgical procedure, involving a larger surface area of the diaphragm, and therefore requires more time to perform, which could lead to more postoperative complications. Indeed, in a large systematic review by Cheng et al. including seven prospective and 59 retrospective studies, prolonged operative time was associated with an increased risk of complications in various surgical fields [39].

Based on the available data from six studies [18, 19, 23–25, 27], there was no significant difference in hospital stay, but this result should be interpreted with caution due to the high heterogeneity of this endpoint. A potential explanation of this observation may rely in the different institutional policies of the contributing studies after cytoreductive surgery. There was no difference in 90-day mortality and ICU stay between the two groups. This observation suggests that despite the higher incidence of major postoperative complications after full-thickness diaphragmatic resection, 90-day mortality and ICU stay are not affected, although it should be noted that ICU stay is only examined in three studies [18, 19, 25] and 90-day mortality in five studies [19, 20, 23, 24, 27], so more data are probably needed to draw a definitive conclusion.

Current literature shows that cytoreductive surgery in advanced ovarian-and colorectal cancer with peritoneal metastasis could lead to a better overall- and disease free survival [10, 40–42]. From the 10 included studies in this meta-analysis [18–27], overall survival was only mentioned in four [18, 22–24] and based on the provided data we were not able to perform a meta-analysis of the overall survival. While Zapardiel et al. [18], Sullivan et al. [24], and Soleymani et al. [23] reported a higher overall survival in the full-thickness diaphragmatic resection group, the results of Singh et al. showed a different pattern in overall survival based on tumour entity: colorectal cancer, low-grade appendiceal mucinous neoplasms and mesothelioma showed a higher overall survival after 40 months in the diaphragmatic stripping group, whereas high grade appendiceal mucinous neoplasms demonstrated a higher overall survival in the full-thickness resection cohort (overall survival diaphragmatic stripping: 30%, 80%, 60%, and 10%, and full-thickness diaphragmatic resection: 20%, 40%, 30%, and 50%, respectively) [22]. In order to be able to draw conclusions about the impact on overall,-and disease-free survival rates between these two surgical techniques, larger and homogeneous clinical trials with complete long-term follow-up data are needed. It is noteworthy that the pooled data presented here come from studies in which mainly patients with ovarian cancer were treated. However, conflicting results are reported in the literature on perioperative short-term outcomes depending on the type of diaphragmatic intervention for other non-ovarian malignancies. For example in the study by Singh et al. diaphragmatic resection was associated with significantly higher rates of adverse events such as pleural effusion, reoperation, in-hospital mortality, and a prolonged hospital stay in mesothelioma and mucinous neoplasms, respectively [22]. On the other hand, Sullivan et al. demonstrated no significantly different perioperative outcomes between diaphragmatic stripping and diaphragmatic resection interventions in a cohort of predominantly GI and hepatobiliary tumors [24].

When interpreting the results, several limitations must be taken into account: firstly, all studies except one were retrospectively conducted. Secondly, the inclusion and exclusion criteria of the studies varied considerably within the monocentric design setting, and most importantly many variables of interest were not provided throughout all eligible studies. In addition, the included studies applied different surgical techniques and suture materials to close the diaphragm defect in full-thickness diaphragmatic resection. Of note, the feasibility of randomized controlled trials is questionable as most cases reported in the literature required full-thickness resection for potential tumor clearance due to the nature of the underlying disease and extent of peritoneal involvement.

## Conclusions

Based on our results, diaphragmatic stripping should be performed during cytoreductive surgery whenever possible, as it allows for rapid postoperative recovery without compromising the oncologic outcome. Ideally, larger trials with homogeneous study protocols and long-term survival analyses are required to validate these findings.

## Supplementary Information

Below is the link to the electronic supplementary material.

Supplementary file1 (DOCX 28 KB)

Supplementary file2 (DOCX 30 KB)
