# Supplementary material for: Diaphragmatic stripping versus full-thickness diaphragmatic resection in cytoreductive surgery: a meta-analysis of the current evidence
Source: Langenbecks Arch Surg. 2025 Jan 25;410(1):50. doi: 10.1007/s00423-025-03611-0 (PMC11762642; doi:10.1007/s00423-025-03611-0)
Supplement: Supplementary file 1 — Supplementary file1 (DOCX 28 KB) [file 423_2025_3611_MOESM1_ESM.docx]

Table suppl. 1 The GRADE Certainty assessment for the significant outcomes

| Outcomes | No. of studies | **No. of included patients** | | SMD/OR (95 % CI) | **Quality assessment** | | | | | Quality |
| --- | --- | --- | --- | --- | --- | --- | --- | --- | --- | --- |
|  |  | Stripping | Resection |  | Risk of bias^a^ | Inconsistency | Indirectness | Imprecision | Publication bias |  |
| Pleural effusion | 9 [18,19,21–27] | 908 | 348 | 0.47 (0.35–0.63) | Not serious | Not serious | No indirectness | No imprecision | NA | Moderate |
| Pneumothorax | 7 [18,21–25,27] | 796 | 304 | 0.52 (0.35–0.78) | Not serious | Not serious | No indirectness | No imprecision | NA | Moderate |
| Severe complications | 5 [19,20,22,23,27] | 607 | 196 | 0.43 (0.30–0.63) | Not serious | Not serious | No indirectness | No imprecision | NA | Moderate |
| Surgery duration (min) | 6 [18,19,21,23–25] | 403 | 239 | -0.90 (-1.63 – -0.17) | Not serious | Serious | No indirectness | No imprecision | NA | Very low |

NA: Not applicable; OR: Odds ratio; SMD: Standardized mean difference a Risk of bias assessed using the ROBINS-I criteria
